# Inhibition of Neovascularization and Inflammation in a Mouse Model of Corneal Alkali Burns Using Cationic Liposomal Tacrolimus

**DOI:** 10.3389/fbioe.2021.791954

**Published:** 2021-12-07

**Authors:** Xueqi Lin, Xuewen Yu, Xiang Chen, Siting Sheng, Jingwen Wang, Ben Wang, Wen Xu

**Affiliations:** ^1^ Eye Center, The Second Affiliated Hospital, Zhejiang University School of Medicine, Hangzhou, China; ^2^ Zhejiang Provincial Key Lab of Ophthalmology, The Second Affiliated Hospital, School of Medicine, Zhejiang Univerity, Hangzhou, China; ^3^ Cancer Institute (Key Laboratory of Cancer Prevention and Intervention, China National Ministry of Education), The Second Affiliated Hospital, Zhejiang University School of Medicine, Hangzhou, China; ^4^ Institute of Translational Medicine, Zhejiang University, Hangzhou, China

**Keywords:** tacrolimus, liposomes, corneal neovascularization, bioavailability, corneal permeability, ocular retention time

## Abstract

Eye drops account for more than 90% of commercialized ophthalmic drugs. However, eye drops have certain shortcomings, such as short precorneal retention time and weak corneal penetration. The requirement of frequent instillation of eye drops also causes poor patient compliance, which may lead to further aggravation of the disease. We aimed to develop a cationic liposome formulation to increase the bioavailability of the therapeutic agent and solve the aforementioned problems. In the present study, we prepared cationic liposomal tacrolimus (FK506) with a surface potential of approximately +30 mV, which could bind to the negatively charged mucin layer of the ocular surface. Our results showed that the content of FK506 in the cornea was increased by 93.77, 120.30, 14.24, and 20.36 times at 5, 30, 60, and 90 min, respectively, in the FK506 liposome group (0.2 mg/ml) compared with the free drug group (0.2 mg/ml). Moreover, FITC-labeled FK506 liposomes significantly prolonged the ocular surface retention time to 50 min after a single dose. In addition, the results of the Cell Counting Kit-8 assay, live and dead cell assay, sodium fluorescein staining, and hematoxylin and eosin staining all indicated that FK506 liposomes had good biological compatibility in both human corneal epithelial cells and mouse eyeballs. Compared with the free drug at the same concentration, FK506 liposomes effectively inhibited vascular endothelial growth factor-induced green fluorescent protein-transduced human umbilical vein endothelial cell migration and tube formation *in vitro*. In a mouse corneal neovascularization model induced by alkali burns, FK506 liposomes (0.2 mg/ml) enhanced corneal epithelial recovery, inhibited corneal neovascularization, and reduced corneal inflammation, and its therapeutic effect was better than those of the commercial FK506 eye drops (1 mg/ml) and the free drug (0.2 mg/ml). Collectively, these results indicate that cationic FK506 liposomes could increase the efficacy of FK506 in the corneal neovascularization model. Therefore, cationic FK506 liposomes can be considered as a promising ocular drug delivery system.

## 1 Introduction

Corneal neovascularization (CoNV), one of the most common causes for reduced corneal transparency and vision loss, can be induced by inflammation and a variety of angiogenic stimuli (Chuang et al., 2019; Chen et al., 2021). At present, among the many therapeutic agents used for the treatment of CoNV caused by alkali burns, anti-inflammatory drugs and anti-vascular endothelial growth factor (VEGF) drugs are the most commonly used regimens (Roshandel et al., 2018). FK506 is a potent immunosuppressant and an effective drug candidate for CoNV therapy; previous studies have reported the anti-angiogenesis and anti-inflammation effects of FK506 in treating corneal alkali burns in mice (Park et al., 2015; Chen et al., 2018; Wu et al., 2019).

Eye drops remain the most common method of administration for the current treatment of eye diseases, accounting for 90% of commercialized ophthalmic drugs (Alvarez-Trabado et al., 2017; Jumelle et al., 2020). Although eye drops are the most non-invasive and the simplest method of administration, due to the complicated anatomical and physiological characteristics of the eye as well as the rapid clearance mechanisms (e.g., tear dilution, blinking, and nasolacrimal duct drainage) on the ocular surface, 95% of these drugs are drained to the whole body through the nasolacrimal duct, and the bioavailability of common eye drops is extremely low (1–5%) (Lin et al., 2021). To achieve therapeutic effects, high concentrations and frequent use of eye drops are usually required to achieve a locally effective drug concentration, and this can result in serious off-target effects and low patient compliance (Urtti, 2006; Mu et al., 2018; Jumelle et al., 2020). Extending the ocular surface residence time and increasing the corneal permeability are the two most common methods of ameliorating drug bioavailability (Jumelle et al., 2020). Previous studies have reported that some FK506 formulations, such as liposomes (Pleyer et al., 1993; Whitcup et al., 1998; Zhang et al., 2010; Dai et al., 2013), micelles (Lin et al., 2019), and niosomes (Li et al., 2014; Zeng et al., 2016), have effectively enhanced the corneal permeability and have been evaluated in animal models of uveitis, dry eye, and corneal transplantation. In addition, previous extensive studies have also demonstrated that for poorly water-soluble drugs, such as FK506, cationic liposomes show potential for enhancing ocular bioavailability (Gai et al., 2018). However, to the best of our knowledge, no previous research has evaluated the potential of cationic FK506 liposomes in the treatment of corneal alkali burns.

In this study, we developed cationic FK506 liposome eye drops using cationic phospholipids 1,2-dioleoyl-3-trimethylammonium-propane (DOTAP) and 1,2-dioleoyl-sn-glycero-3-phosphoethanolamine (DOPE). This study aimed to demonstrate that in comparison with commercial FK506 eye drops and the free drug, FK506 liposomes showed improved therapeutic effects on CoNV and corneal inflammation in a mouse corneal alkali burn model. Moreover, we evaluated the biocompatibility of cationic FK506 liposomes, their inhibitory effects on angiogenesis *in vitro* and *in vivo*, and the improvement in corneal permeability and ocular retention time.

## 2 Materials and Methods

### 2.1 Materials

DOTAP was acquired from AVT Pharmaceutical Tech Co., Ltd (Shanghai, China). DOPE was obtained from Sigma. 1,2-distearoyl-sn-glycero-3-phosphoethanolamine-N-[methoxy(polyethyleneglycol)-2000] (DSPE-PEG2000) was provided by Shanghai Yuanye Biological Technology Co., Ltd. FK506 was purchased from Selleck (Shanghai, China). Solvents, such as chloroform, methanol, and acetonitrile, were supplied by the materials and chemicals procurement management platform of Zhejiang University.

### 2.2 Preparation of FK506 Liposomes

FK506 liposomes were prepared using the membrane hydration method according to the published procedure (Mendez et al., 2014; Wu et al., 2021). Briefly, DOTAP, DOPE, cholesterol, DSPE-PEG2000 (at a ratio of 4:4:4:1) and 2 mg FK506 were dissolved in chloroform, and the solvent was evaporated on a rotary evaporator to form a semi-transparent lipid film. Then, the film was hydrated with PBS, sonicated for 40 min, and filtered through a 0.2-μm polycarbonate membrane extrusion filter to further homogenize the particle size. The DSPE-PEG2000 was replaced with FITC-PEG2000-DSPE for the FITC-labeled liposome synthesis.

### 2.3 Characterization of FK506 Liposomes

#### 2.3.1 Transmission Electron Microscopy

The liposomes were placed on a copper mesh and then negatively stained with ammonium molybdate. The morphology and structure of the FK506 liposomes were observed using TEM (JEM-1400, JEOL, Japan) at the acceleration voltage of 80 kV.

#### 2.3.2 Size Distribution and Zeta Potential

The particle size, zeta potential, and polydispersity (PDI) of FK506 liposomes and blank liposomes were determined using a Malvern Nano-ZSE laser particle size analyzer. The liposomes were diluted 100 times, followed by gentle vibration before measurement.

#### 2.3.3 *In vitro* Drug Release Profile and Entrapment Efficiency

The release profile of FK506 liposomes was measured as follows. Briefly, an 8-ml volume of FK506 liposomes was transferred to a dialysis bag and then submerged in 30 ml of 1% sodium dodecyl sulfate solution for drug release analysis. At the indicated time points, 1 ml of dialysate was taken out and freeze dried. Later, the freeze-dried powder was re-dissolved in 0.4 ml of methanol, and the drug concentration was measured by HPLC (Agilent 1100, USA, FK506 210 nm).

The HPLC conditions were as follows: C18 column (250 mm × 4.6 mm 4 micron, Phenomenex); the mobile phase included acetonitrile and 0.1% phosphoric acid (80:20, v:v); flow rate: 1 ml/min; and column temperature: 50°C. The EE was calculated as follows: EE (%) = amount of FK506 entrapped/total amount of FK506 × 100%.

### 2.4 Cells

Green fluorescent protein-transduced human umbilical vein endothelial cells (HUVEC-GFPs) and human corneal epithelial cells (HCECs) were maintained in DMEM medium (Corning, USA) containing 1% penicillin, 1% streptomycin, and 10% fetal bovine serum (FBS) in a 37°C incubator supplied with 5% CO_2_.

#### 2.4.1 Cell Counting Kit-8 Assay

Briefly, 5,000 HCECs were inoculated per well in a 96-well plate. After culturing for 24 h, different concentrations of the free drug and FK506 liposomes (0, 3.91, 7.81, 15.63, 31.25, and 62.5 μg/ml) were added into each well for another 24-h incubation. Then, the HCECs were gently washed with PBS and replaced with 100 μL of medium containing 10 μL of CCK-8 liquid (Dojindo, Japan) for CCK-8 analysis. After incubating for another 2 h, the absorbance values were measured at 450 nm on a microplate reader (Molecular Devices Spectramax M5, USA).

#### 2.4.2 Live and Dead Cell Assay

HCECs were inoculated in a 24-well plate at a density of 1 × 10^4^ cells/well and cultured overnight. Then, the free drug and the FK506 liposomes were added at different concentrations (0, 5, 25, and 35 μg/ml) and incubated for 24 h. Subsequently, the drug solution was removed, and the cells were washed 3 times with PBS. After that, the cells were stained using a Calcein-AM/PI double staining kit (Yeason, China) (Calcein-AM, 0.67 μM; PI, 1.5 μM) and incubated at 37°C for 15 min in the dark. Finally, an inverted fluorescence microscope (Zeiss, Germany) was used to record the images.

#### 2.4.3 Cell Model

HUVEC-GFP cells were cultivated according to the conditions mentioned above. *In vitro* angiogenesis assays were carried out by adding a high concentration of recombinant human VEGF-165 (rhVEGF, PeproTech, USA) to the culture medium to simulate the environment of neovascularization.

#### 2.4.4 Wound Healing Assay

HUVEC-GFP cells were seeded in a 12-well plate at a density of 1 × 10^4^ cells/well and were adhered overnight. Subsequently, a 200-μL pipette tip was utilized to produce uniform scratches. Then, serum-free DMEM containing 100 ng/ml VEGF was added with 5 μg/ml of the free drug or FK506 liposomes and incubated with the cells for another 48 h. The migration profile was recorded by an inverted fluorescence microscope at 24 and 48 h, and the widths of the scratches were measured by ImageJ software (NIH, USA).

#### 2.4.5 Transwell Migration Assay

HUVEC-GFP cells (1.5 × 10^4^) were resuspended in serum-free DMEM containing different concentrations of FK506 liposomes and the free drug (0, 5, 15, and 25 μg/ml) and seeded in the upper chamber of Transwell inserts (BD Bioscience, USA). The lower chamber was a medium (600 μL) containing 10% FBS as a chemoattractant. After incubating for 24 h, the cells were dyed with a mixed solution of 4% paraformaldehyde and 0.05% crystal violet for 15 min and then thoroughly washed with PBS solution. Then, the cells in the upper chamber were gently wiped off, and the cells in the lower chamber were counted under a light microscope.

#### 2.4.6 Tube Formation Assay

Briefly, 250 μL of Matrigel (Corning, 356230) was carefully smeared in a pre-cooled 24-well plate and incubated at 37°C to form a gel. Later, 2.3 × 10^5^ HUVEC-GFP cells were suspended in the culture medium, supplemented with 200 ng/ml rhVEGF and different concentrations of FK506 liposomes or the free drug, and seeded on the surface of the Matrigel. At 6 h, tube formation was captured using an inverted fluorescence microscope and analyzed using ImageJ software.

### 2.5 Animals

All C57BL/6 mice (6–8 weeks, 20–25 g, male) were purchased from Shanghai SLAC Laboratory Animal Co., Ltd. New Zealand rabbits weighing from 2 to 2.5 kg were acquired from the Experimental Animal Center of Zhejiang Academy of Medical Sciences (Hangzhou, China).

#### 2.5.1 Ethics Statement

The animal experiments were carried out following the “Statement on the Use of Animals in Ophthalmology and Vision Research” described by the Association for Research in Vision and Ophthalmology. The research protocols were approved by the Animal Ethics Committee of the Second Affiliated Hospital of Zhejiang University School of Medicine (batch number: [2021] No. [057]).

#### 2.5.2 Ocular Surface Stimulation Test

FK506 liposome eye drops (0.2 mg/ml, 10 μL) were locally administered four times a day for 14 days. Mice treated with normal saline (NS) served as the control group. On day 14, the eye irritation was evaluated using a slit lamp microscope (66 Vision-Tech, China) and corneal sodium fluorescein staining; then, the eyeballs were removed for hematoxylin and eosin (H&E) staining.

#### 2.5.3 Biocompatibility Evaluation of FK506 Liposomes *in vivo*

The mice were sacrificed at the end of the treatment. For each mouse, the blood was collected and centrifuged at 3,000 g for 15 min, and the supernatant was taken for liver and kidney function analysis. In addition, the heart, liver, spleen, lung, and kidney were collected and subjected to H&E staining for the toxicity assessment.

#### 2.5.4 Mouse Model of Corneal Alkali Burns

Mice corneal alkali burn model was established as previously described (Anderson et al., 2014). The mice were generally anesthetized via intraperitoneal injection of 0.3% pentobarbital sodium and locally anesthetized using proparacaine hydrochloride eye drops (Alcon-Couvreur, Belgium). The 2-mm round filter papers were soaked in 1M NaOH solution for 20 s, placed in the center of the mice corneas for 40 s, and then quickly removed. Finally, 20 ml of NS was used to rinse the ocular surface. On the first day after surgery, all animals were randomly divided into four groups: the NS group (*n* = 6), the commercial FK506 eye drop group (FK 1 mg/ml, *n* = 6), the free drug group (0.2 mg/ml) (*n* = 6), and the FK506 liposome group (0.2 mg/ml) (*n* = 6). Treatment for each mouse was administered four times a day (10 μL each time) for 14 days.

#### 2.5.5 Clinical Assessment

On days 1, 3, 7, and 14, the corneas of the mice from different groups were observed, photographed, and scored according to the previous scoring system (Han et al., 2020b; Zhong et al., 2021). Briefly, the corneal turbidity was scored from 0 to 4 points, and the CoNV area and the size of the blood vessels were scored from 0 to 3 points. Additionally, each cornea was stained with fluorescein sodium ophthalmic strips for examination of the corneal epithelial defect. ImageJ software was used to quantify the vessel lengths, CoNV areas, and areas of corneal epithelial defect.

#### 2.5.6 H&E Staining and Immunohistochemistry Staining

The mouse eyeballs were enucleated, fixed in 4% paraformaldehyde overnight, embedded in paraffin, and then prepared into 4-μm-thick paraffin sections. H&E staining was carried out, and immunohistochemistry (IHC) staining was conducted using anti-α-smooth muscle actin (Wuhan Sanying Biotechnology; dilution 1:2,000).

#### 2.5.7 Immunofluorescence Staining

After fixing in 4% paraformaldehyde for 1 h, the intact corneas were carefully peeled off and blocked for 2 h. Then, the corneas were incubated with rabbit anti-mouse CD31 antibody (Abcam; dilution 1:50) at 4°C overnight and then incubated with Alexa Fluor 555-conjugated goat anti-rabbit secondary antibodies (Invitrogen; dilution 1:500) for 1 h in the dark. Finally, the corneas were radially cut, flat mounted, and observed under a fluorescence microscope.

#### 2.5.8 Quantitative RT-PCR

The total RNA of the cornea was extracted using TRIzol reagent (Accurate Biotechnology, China). Then, reverse transcription and real-time PCR were carried out according to the operating steps provided in the instructions. The relative expressions of the target genes, such as VEGF-A, VEGF-R3, interleukin (IL)-6, and matrix metalloproteinase (MMP)-9, were determined using the 2^−∆∆Ct^ method, and GAPDH was adopted as the housekeeping gene.

#### 2.5.9 Retention of FK506 Liposomes on the Ocular Surface

Three types of fluorescent eye drops (FITC dye solution, free-drug solution, and FITC-labeled FK506 liposomes, 5 μL) were dripped into the eyes of the mice after they were anesthetized. A Xenogen IVIS Lumina imaging system (Perkin Elmer, Akron, OH, USA) was used to capture images of the eyes every 5 min, and the percentage of residual fluorescence intensity was calculated as the proportion of the fluorescence intensity at the corresponding time point to the initial fluorescence intensity.

#### 2.5.10 FK506 Content in the Cornea/Aqueous Humor

Briefly, 75 μL of three FK506 preparations (0.02% FK506 liposome eye drops, 0.02% free-drug solution, and 0.1% commercial FK506 eye drops) was added to the eyes of the rabbits, respectively. The rabbits were euthanized at 5, 30, 60, and 90 min after administration, the corneas were collected, and 150 μL of aqueous humor was harvested. After cutting into pieces, the cornea was suspended in two volumes of NS containing high-strength ceramic beads and then fully broken. Then, three volumes of acetonitrile were added to extract the drug from the cornea. As for the aqueous humor, one volume of acetonitrile was added to the sample to extract the drug. Finally, the samples were centrifuged at 10,000 rpm for 10 min, and 200 μL of supernatant was used for FK506 concentration measurement using a mass spectrometer (Agilent Technologies, USA).

The HPLC conditions of the LC/MS/MS were as follows: Zorbax SB C18 column (150  mm × 2.1  mm, 3.5 µm); the mobile phases included phase A and phase B (phase A contained 5 mmol sodium acetate and 0.1% formic acid, and phase B was methanol); the precursor was 821.5 m/z; the ion product was 786.4; and the flow rate was 0.3 ml/min.

### 2.6 Statistical Analysis

All data were expressed as means ± standard deviations (SDs). The statistical differences between groups were determined using two-tailed Student’s t test or one-way analysis of variance (ANOVA). A *p*-value of <0.05 was considered statistically significant.

## 3 Results and Discussion

### 3.1 Characterization of FK506 Liposomes

#### 3.1.1 Morphology, Size Distribution, and Zeta Potential

**FIGURE 1:**
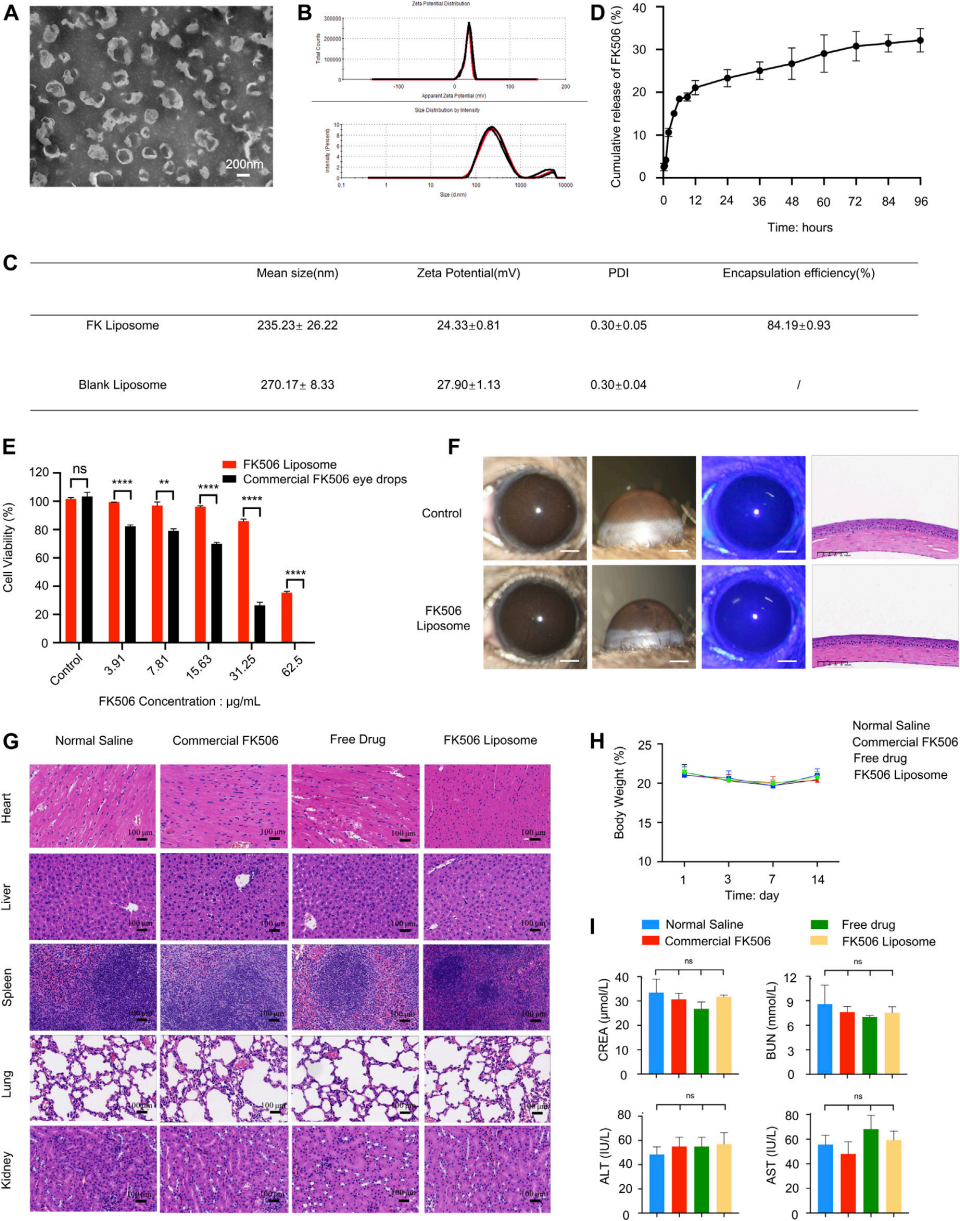
Characterization and biocompatibility evaluation of FK506 liposomes. **(A)** TEM image of FK506 liposomes. Scale bar: 200 nm. **(B)** Zeta potential and size distribution of liposomes revealed by dynamic light scattering. **(C)** Characterization of FK506 liposomes and blank liposomes. **(D)** *In vitro* release profile of FK506 liposomes (*n* = 4). **(E)** CCK-8 assay of the HCECs exposed to free-drug or FK506 liposomes (*n* = 3). **(F)** *In vivo* biocompatibility evaluation of FK506 liposomes. The corneas were examined with a slit lamp microscope (scale bar: 1 mm), corneal fluorescein staining (scale bar: 1 mm), and H&E staining (scale bar: 100 μm) after 14 days of treatment with NS or FK506 liposomes (0.2 mg/ml). **(G)** Topical application of FK506 liposomes neither influenced the function of the main organs *in vivo* (scale bar: 100 μm) nor changed the **(H)** body weight of the mice in the four groups during the 14-days treatment period. **(I)** Toxicity studies of liver function (ALT, AST) and kidney function (UA, BUN) in mice after receiving commercial FK506 eye drops (1 mg/ml), free drug solution (0.2 mg/ml), or FK506 liposomes (0.2 mg/ml) for 14 consecutive days (*n* = 3). The data was presented as mean ± SD, two-tailed Student’s t test and one-way ANOVA, *p < 0.05, **p < 0.01, ***p < 0.001, and ****p < 0.0001; ns denotes no significance.

The TEM image (Figure 1A) showed that the liposomes were nearly spherical nanostructures with a particle size of approximately 200 nm. Further dynamic light scattering results (Figures 1B,C) indicated that the average hydrodynamic diameter of FK506 liposomes and blank liposomes were 235.23 ± 26.22 nm and 270.17 ± 8.33 nm, respectively. And the particle dispersion index of the two liposomes were 0.30 ± 0.05 and 0.30 ± 0.04, indicating that the particle size of the liposomes was uniformly dispersed. Moreover, the zeta potential of FK506 liposomes and blank liposomes were approximately +24.33 ± 0.81 mV and +27.90 ± 1.13 mV, respectively (Figure 1C).

Previous literature has shown that a small particle size can enhance the permeability of the cornea, hence increasing the bioavailability of the therapeutic agent. The small particle size and uniform spherical structure can also ensure low irritation to the ocular tissues when used as an ophthalmic drug delivery system (Gai et al., 2018). Furthermore, the positive charge of the cationic liposomes not only provides an effective repulsive force between the liposomes and the negatively charged mucins on the ocular surface, therefore significantly prolonging the drug retention time and facilitating the uptake of liposomes by the ocular surface cells, but also enhances the stability of the liposomes when used as a drug delivery system.

#### 3.1.2 *In vitro* Drug Release Profile and Entrapment Efficiency

Figure 1D shows the 96-h *in vitro* cumulative release curve of FK506 liposomes. FK506 liposomes released approximately 28.14% of the total amount of the drug in 96 h *in vitro*; the drug was released more quickly in the first 6 h (approximately 16.16% was released in the first 6 h). Moreover, the average EE of FK506 liposomes was 84.19 ± 0.93% (Figure 1C) (measured through three batches of FK506 liposomes).

The rapid release rate of FK506 liposomes in the early stage provides a sufficient drug dose to inhibit corneal inflammation in the initial stage of CoNV. Meanwhile, the slower, sustained release in the later stage helps to release the drug for a long period of time to inhibit the growth of CoNV. Since FK506 is a hydrophobic drug that is encapsulated in the lipid bilayer of liposomes, the slow release of FK506 can be ascribed to the high affinity of FK506 to the hydrophobic part of the formulation. The *in vitro* sustained-release curve showed that FK506 liposomes possessed sustained-release properties after being encapsulated by liposomes.

### 3.2 Biocompatibility Evaluation of FK506 Liposomes

#### 3.2.1 Cytotoxicity Evaluation *in vitro*

At all concentrations, the viability of the cells from the FK506 liposome group was significantly higher than that of cells from the free-drug group (Figure 1E). Even at an FK506 concentration of 31.25 μg/ml, the cell viability of HCECs in the FK506 liposome group was still more than 80% (86.02 ± 2.52%), while the cell viability of the free-drug group at the same concentration was only 26.48 ± 3.55% (*p* < 0.0001). Similarly, live and dead cell staining was conducted; red and green represented dead cells and live cells, respectively. At the same drug concentration, the number of live cells in the visual field in the FK506 liposome group was markedly higher than that in the free-drug group (Supplementary Figure 1).

Since cationic liposomes have certain cytotoxicity, it is necessary to evaluate the biocompatibility of cationic FK506 liposomes beforehand. Both the CCK-8 assay and Calcein-AM/PI staining demonstrated that the cationic FK506 liposomes had good biosafety, and the toxicity of cationic FK506 liposomes to HCECs was much less than that of the free FK506 at the same drug concentration, indicating that the cytotoxicity of FK506 could be significantly reduced after liposomal encapsulation.

#### 3.2.2 Ocular Surface Stimulation Test

After 14 days of topical administration of FK506 liposomes, the results of the slit lamp evaluation indicated that the corneas of the mice were smooth and transparent, and no hyperemia, inflammation, or neovascularization was found. Moreover, the sodium fluorescein images showed no green staining, indicating that there were no defects in the corneal epithelium during the FK506 liposome treatment. In addition, H&E staining indicated that the anatomical structures of the mouse corneas were complete and neatly arranged, and no vascular lumens or inflammatory cells were observed, similarly to the corneas of the normal mice. These results demonstrated that FK506 liposomes had no potential negative effects on mouse corneas, indicating good ocular biocompatibility. These results are shown in Figure 1F.

#### 3.2.3 Biocompatibility Evaluation of FK506 Liposomes *in vivo*

The mice treated with NS, commercial FK506 eye drops, the free drug, and FK506 liposomes did not exhibit significant histological differences (Figure 1G). In addition, the body weight of the mice remained stable during the 14 days, with no significant differences between the four groups (Figure 1H). Further biochemical tests showed that the liver functions (alanine aminotransferase, ALT; aspartate aminotransferase, AST) and kidney functions (creatinine, CREA; blood urea nitrogen, BUN) of the mice were all within the normal ranges after receiving different preparations for 14 days (Figure 1I).

Due to the narrow therapeutic window of FK506, long-term use of high-concentration FK506 may cause severe off-target, systemic effects, such as liver and kidney toxicity and neurotoxicity (Liu et al., 2019). Therefore, the aforementioned results all indicate that FK506 liposomes are superior with regard to biosafety.

### 3.3 The Inhibitory Effects of FK506 Liposomes on Vascular Endothelial Growth Factor-Induced Angiogenesis *in vitro*

#### 3.3.1 Wound Healing Assay and Transwell Migration Assay

**FIGURE 2:**
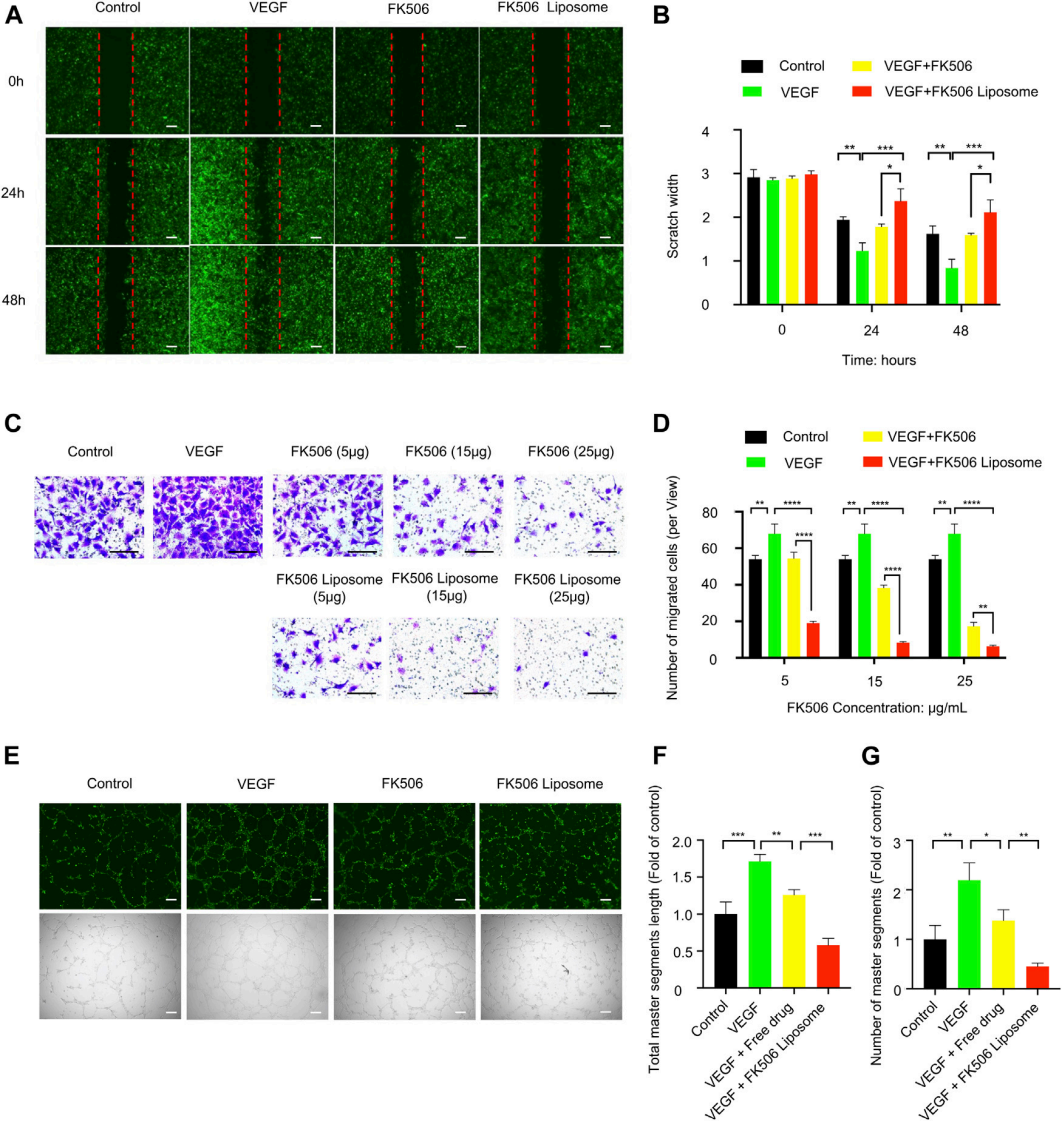
*In vitro* evaluation of FK506 liposomes in the inhibition of angiogenesis. **(A)** FK506 liposomes inhibited the migration of HUVEC-GFP cells by wounding healing assay (scale bar: 200 μm, *n* = 3). **(B)** Quantitative analysis of the scratch widths. **(C)** Different concentrations of FK506 liposomes inhibited the migration of HUVEC-GFP cells by transwell migration assay (scale bar: 100 μm, *n* = 3). **(D)** Quantitative analysis of the number of migrated cells in each view. **(E)** Tube formation activity of HUVEC-GFP cells. Representative fluorescence and bright-field images of HUVEC-GFP cells after co-incubation with 15 μg/ml FK506 or FK506 liposomes for 6 h (scale bar: 200 μm, *n* = 3). Quantitative analysis of **(F)** total master segment length and **(G)** number of master segments.

A wounding healing assay was performed to assess the influence of the free drug and FK506 liposomes at the same concentration on the migration property of HUVEC-GFPs cells *in vitro*. The VEGF treatment (100 ng/ml) induced the surrounding HUVEC-GFP cells to crawl toward the center of the scratch, almost filling the gap within 48 h, showing the smallest scratch width in all treatment groups (Figure 2A). However, both FK506 liposomes and the free drug significantly delayed the migration of HUVEC-GFP cells. Notably, compared with the free drug at 5 μg/ml, FK506 liposomes significantly reduced the migration of HUVEC cells at the same concentration (*p* < 0.05) (Figure 2B). The findings of the transwell assay revealed the same tendency (Figure 2C). FBS served as a chemical attractant to promote the migration of HUVECs to the lower chamber of transwell inserts. Compared with the NS, VEGF, and free-drug groups, the number of vascular endothelial cells that migrated to the underside of the transwell membrane was significantly reduced in the FK506 liposome treatment group at all drug concentrations (Figure 2C). The migration rates of HUVECs inhibited by FK506 liposomes were 27.94, 12.25, and 9.31% at FK506 concentrations of 5, 15, and 25 μg/ml, respectively (Figure 2D).

#### 3.3.2 Tube Formation Assay

The tube formation assay indicated that the formation of capillary networks was more abundant in the 200 ng/ml VEGF induction group and that fewer disconnected network tubes were observed in the free-drug and FK506 liposome treatment groups (Figure 2E). In addition, the quantitative analysis found that compared with the free-drug group, at the same concentration, FK506 liposomes were more effective in inhibiting the formation of the capillary networks. After incubation with 15 μg/ml FK506 liposomes for 6 h, tubes showed a shorter total master segment length (*p* < 0.001) and a smaller number of master segments (*p* < 0.01) (Figures 2F,G). Additionally, the result of the tube formation assay was consistent with those of the migration assays of vascular endothelial cells *in vitro*.

Blood vessel formation is induced by many factors, such as hypoxia and inflammation, and followed by the recruitment of inflammatory cells, production of angiogenic factors, basement membrane degradation, and proliferation, migration, and tube formation of vascular endothelial cells, etc. (Nowak-Sliwinska et al., 2018; Chen et al., 2021). FK506 liposomes inhibited the specific stages of angiogenesis induced by rhVEGF_165_, including the migration and tube formation of HUVEC-GFP cells. Among these stages, migration is one of the early steps of angiogenesis (Nowak-Sliwinska et al., 2018), and tube formation indicates the maturation of migrated vascular endothelial cells (Han et al., 2020b). The results mentioned above implied that FK506 liposomes had a stronger inhibitory effect on angiogenesis, which might be attributed to cationic liposomes increasing the intracellular uptake of FK506.

### 3.4 Anti-Corneal Neovascularization Effect of FK506 Liposomes *in vivo*

#### 3.4.1 Inhibition of Neovascularization

**FIGURE 3:**
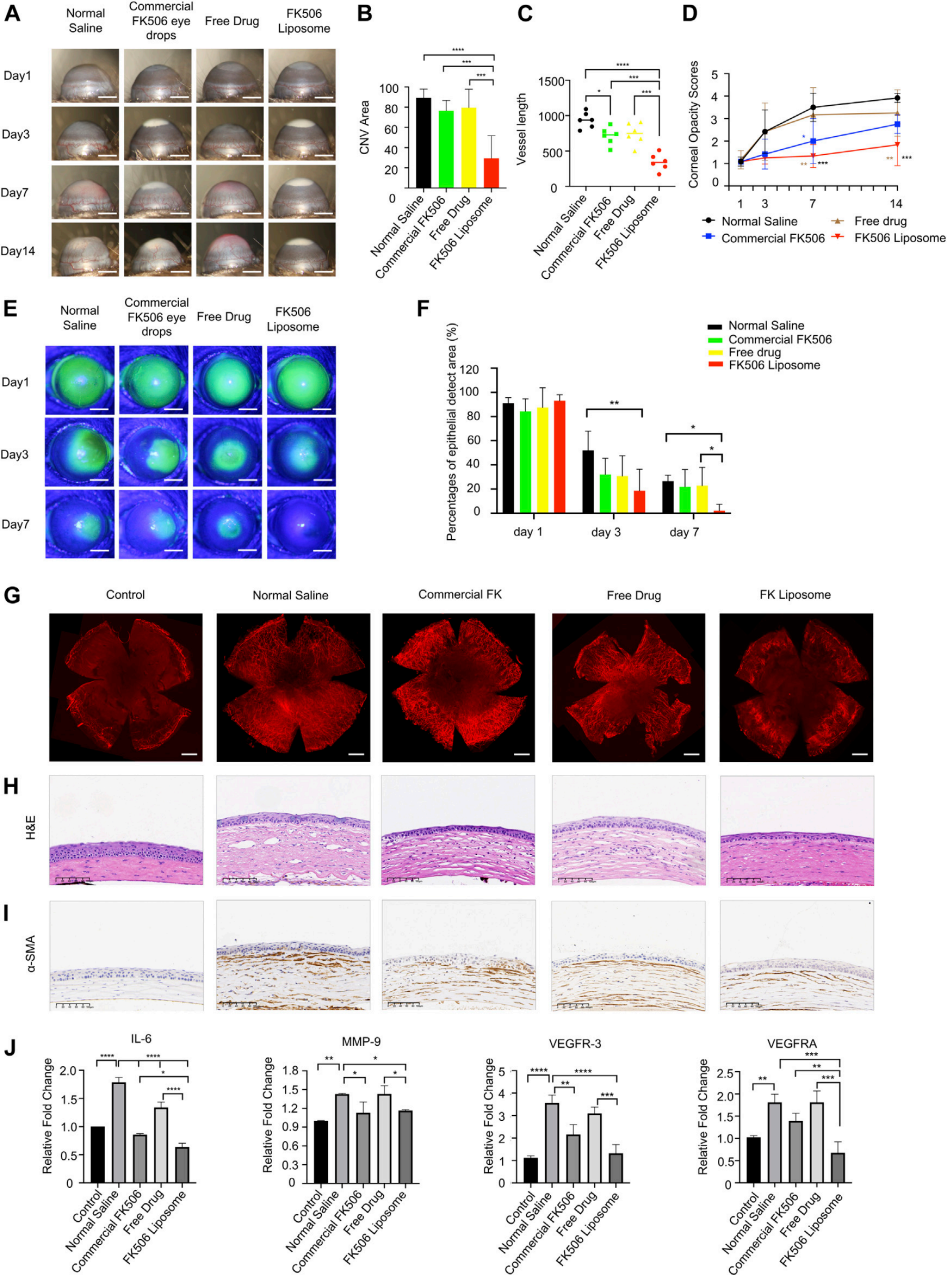
*In vivo* therapeutic effect evaluation of FK506 liposomes in mouse corneal alkali burn model. **(A)** Slit-lamp examination of CoNV on the 1st, 3rd, 7th, and 14th days after corneal alkali burn. Scale bar: 1 mm. **(B)** Quantification of the CoNV area (*n* = 6). **(C)** Quantitative analysis of the corneal vessel length (*n* = 6). **(D)** The corneal opacity score on the 14th day of treatment (*n* = 6). **(E)** The corneal sodium fluorescein staining areas in each treatment group on days 1, 4, and 7 (scale bar: 1 mm) and their **(F)** quantitative analysis (*n* = 6). **(G)** CD31 vascular endothelial marker immunofluorescence staining on day 14. Scale bar: 500 μm. **(H)** H&E staining and **(I)** α-SMA IHC staining of corneal sections after treatment with various formulations for 14 days. Scale bars: 100 μm. **(J)** The expressions of vascular endothelial growth factors (VEGF-A, VEGFR-3) and inflammatory factors (IL-6, MMP-9) in the corneas of each treatment group on the 14th day (*n* = 3). The data was presented as mean ± SD, one-way ANOVA, *p < 0.05, **p < 0.01, ***p < 0.001, and ****p < 0.0001.

On day 14, neovascularization in the NS group and free-drug group formed and almost invaded the center of the cornea (Figure 3A). Dense vessels around the entire eyeball were observed from the limbus, and the CoNV areas of the two groups accounted for 89.41 ± 8.58% and 79.59 ± 18.17% of the entire cornea, respectively (Figure 3B). In addition, anterior chamber hemorrhage was also observed in some mice in the NS group. Moreover, the average CoNV area was 76.64 ± 10.03% in the commercial FK506 eye drop group. In contrast, the FK506 liposome group showed only moderate neovascularization, which was mainly confined around the limbus, and the blood vessels were relatively sparse (Figure 3A). Compared with the NS group, the commercial FK506 eye drop group, and the free-drug group, the CoNV area was reduced by 59.97% (*p* < 0.0001), 47.2% (*p* < 0.001), and 50.15% (*p* < 0.001) in the FK506 liposome group (Figure 3B), respectively. In addition, the lengths of the blood vessels increased significantly with time. On day 14, the vessel lengths in the NS group (937.57 ± 111.66), commercial FK506 eye drop group (711.89 ± 124.48), free-drug group (745.24 ± 152.80), and FK506 liposome group (342.90 ± 116.85) were quantitatively analyzed (Figure 3C). The results showed that both the commercial FK506 eye drops and FK506 liposomes effectively reduced the vessel lengths; the effect of FK506 liposomes was better (*p* < 0.0001 vs. NS; *p* < 0.001 vs. free drug; *p* < 0.001 vs. commercial FK506 eye drops). Compared with the NS group, mice treated with FK506 liposomes showed a reduction in vessel length of approximately 63.43% (Figure 3C).

Meanwhile, the corneas were also stained with a vascular endothelial cell marker (CD31) to more intuitively study the CoNV area at the end of the 14th day (Figure 3G). Similar to the aforementioned results, after administration of FK506 liposomes, the CoNV area and vessel length were significantly reduced compared with the other three groups (Figure 3G; Supplementary Figure 2), indicating that FK506 liposomes were more effective in delivering drugs and inhibiting the formation of new blood vessels compared with the free drug and commercial FK506 eye drops.

Bakunowicz-Łazarczyk and Urban (2016) showed that in the acute phase of alkali burns, anti-inflammation, anti-angiogenesis, and enhancing epithelial healing are the key aspects in clinical treatments. The new blood vessels gradually grew out on days 3 and 7 and reached the peak on day 14. New vessels can block and diffract the penetration of light through their physical presence and can further damage the structural integrity of the cornea (Sharif and Sharif, 2019). The significant reduction in angiogenesis after FK506 treatment was essential for maintaining optimal vision and protecting the eyes from infection and structural damage. One of the treatment methods for CoNV is to begin anti-angiogenesis at an early stage and stop the formation of neovascularization, while the other is intended to trigger vascular degeneration by inducing the reversal of immature blood vessels. FK506 may act via these two aspects to inhibit the formation of CoNV (Sharif and Sharif, 2019). The therapeutic effect of FK506 liposomes on CoNV was associated with the preocular retention time and the amount of drug entering the cornea. The aforementioned findings further confirmed that FK506 liposomes had a greater effect on reducing CoNV.

#### 3.4.2 Corneal Turbidity Scores

After the mouse model of alkali burns was established, the corneal stroma developed edema, and the corneas lost transparency over time. On days 7 and 14 after treatment, the corneal turbidity score of the cationic FK506 liposome group was markedly lower compared with those of the NS group (day 7, 1.33 ± 0.52 vs. 3.50 ± 0.63, *p* < 0.001; day 14, 1.83 ± 0.93 vs. 3.92 ± 0.20, *p* < 0.001) and the free-drug group (day 7, 1.33 ± 0.52 vs. 3.17 ± 1.21, *p* < 0.01; day 14, 1.83 ± 0.93 vs. 3.25 ± 1.04, *p* < 0.01), indicating that the transparency of the cornea gradually improved after treatment with FK506 liposomes (Figure 3D). In contrast, the corneal transparency in the NS group gradually deteriorated over time. The corneal transparency of the commercial FK506 eye drop group was significantly improved on day 7 compared with that of the NS group (*p* < 0.05). However, no significant difference between the two groups was detected on day 14. Similarly, mice treated with the free drug showed no difference in corneal opacity compared with the NS group on day 14, and the internal structure of the anterior chamber was still not clearly visible.

The cornea is an avascular structure, which is an essential element for obtaining optimal vision (Qazi et al., 2010; Kather and Kroll, 2014; Nicholas and Mysore, 2021). In the case of inflammation and hypoxia, the expressions of angiogenesis factors are up-regulated, and CoNV is formed, leading to a vicious circle of corneal opacity and chronic inflammation. Except for small corneal scrapes, the damaged cornea is unable to return to its original transparency. After a corneal injury, when the corneal stromal cells transform into activated fibroblasts, the reflectivity of the corneal stromal cells is increased, thereby enhancing the turbidity of the cornea (Zahir-Jouzdani et al., 2019). In addition to causing severe tissue damage, corneal alkali burns can also cause the production of many growth factors and inflammatory cytokines, which promote corneal opacity after injury. The results of the present study showed that treatment with FK506 liposomes could help restore corneal optical transparency and vision after alkali burns.

#### 3.4.3 Corneal Epithelial Defect Area

Figure 3E illustrates the typical images of corneal epithelial defects on days 1, 3, and 7 of alkali burn treatment. Sodium fluorescein staining showed that on day 1, corneal surfaces were extensively stained in all groups, indicating that the corneal epithelia in all groups were severely damaged. On day 3, the area of the corneal epithelial defects in the FK506 liposome group (18.67 ± 17.82%) was significantly smaller than that in the NS group (52.00 ± 15.86%) (*p* < 0.01) (Figure 3F). However, there was no difference in sodium fluorescein staining areas among the free-drug group (30.83 ± 16.76%), commercial FK506 eye drop group (32.17 ± 13.26%), and NS group. On day 7, the areas of corneal epithelial defects in the free-drug group (22.83 ± 15.15%) and commercial FK506 eye drop group (21.83 ± 14.28%) were similar to that in the NS group (26.67 ± 4.80%) (Figure 3F). However, quantification results on day 7 showed that the corneal epithelial defects in the FK506 liposome treatment group (2.17 ± 5.31%) were markedly smaller than those in the NS group and the free-drug group (*p* < 0.05 for both), indicating that FK506 liposomes exhibited the fastest wound healing effect and could significantly improve corneal epithelial healing after alkali burns.

Furthermore, CoNV is also closely related to corneal epithelial defects. When the cornea is injured, the normal differentiation of limbal stem cells is disturbed; metaplasia and keratosis will occur in the corneal epithelium, leading to transparency loss and subepithelial CoNV (Kather and Kroll, 2014). In recent studies, the anti-angiogenic effect of the corneal epithelium and its role in the prevention of CoNV have been widely accepted (Roshandel et al., 2018). After alkali burns, the structure of the cornea is destroyed, thereby promoting the entrance of pathogenic factors into the deeper layers of the cornea. After FK506 treatment, the rapid recovery of the corneal epithelium can help rebuild the corneal structure and prevent further invasion of inflammatory mediators (Chowdhury et al., 2013). Therefore, the acceleration of corneal epithelialization in the FK506 liposome group could strengthen corneal healing after alkali burns.

### 3.5 H&E Staining and α-smooth Muscle Actin Staining

As shown in Figure 3H, the most severe corneal stroma edema was found in the NS group, and this group’s corneal thickness was also the thickest among all groups. Additionally, the corneal stromata in the NS group were loosely arranged, and there were many neovascular cavities and inflammatory cells infiltrated. The corneal thickness in the free-drug group was still very high, while the vascular lumens and inflammatory cell infiltration were significantly reduced, indicating that local drug treatment helped restore the normal structure of the cornea. However, the corneas treated with commercial FK506 eye drops and FK506 liposomes only had mild edema, and the central corneal thickness was significantly reduced, showing relatively normal stromata. In addition, the numbers of neovascular lumens and infiltrated inflammatory cells were also lower than in the other two groups. The results of α-SMA IHC staining showed that compared with the other three groups, the FK506 liposome group had a weaker α-SMA staining intensity (Figure 3I).

After an alkali burn, the corneal epithelium is severely damaged, and the corneal stroma has edema. The restoration of the normal anatomical structure of the cornea is the optimal result, and it can promote the restoration of vision. The microscopic morphological changes of the cornea after the FK506 liposome treatment indicated that liposomes effectively reduced the degree of inflammation and inhibited the formation of CoNV after alkali burns. Furthermore, α-SMA is a vascular smooth muscle marker produced by fibroblasts that can locate mature blood vessels (Chen et al., 2021). After alkali burns, fibroblasts produce α-SMA in the process of scar formation, leading to corneal fibrosis and severe pathological corneal opacity, which greatly affects a patient’s corneal transparency (Joung et al., 2020). Therefore, inhibiting the differentiation of fibroblasts into myofibroblasts after a corneal alkali burn is an important aspect of treatment. The results of both H&E staining and α-SMA staining showed that the administration of FK506 liposomes helped to restore the normal structure of the cornea and reduce the degree of corneal fibrosis.

### 3.6 Evaluation of Corneal Inflammation and Angiogenesis-Related Factors Using qRT-PCR

To further explore the therapeutic mechanisms of FK506 liposomes in CoNV and corneal inflammation caused by alkali burns, qRT-PCR was used to study the expressions of several inflammatory and angiogenesis-related cytokines at the mRNA level on day 14. The corneas in the NS group expressed high levels of angiogenic factors VEGF-A and VEGFR-3 and inflammation-related cytokines IL-6 and MMP-9 (*p* < 0.01 vs. controls) (Figure 3J). Compared with the other three treatment methods, FK506 liposomes significantly reduced the expression of VEGF-A (*p* < 0.0001 vs. NS; *p* < 0.001 vs. the free drug; *p* < 0.01 vs. commercial FK506 eye drops). Moreover, both the commercial FK506 eye drops and the FK506 liposomes effectively reduced the expression of MMP-9 (*p* < 0.05). Furthermore, all treatment groups exhibited reduced expression of IL-6; the reduction was the most significant in the FK506 liposome group (*p* < 0.0001 vs. NS; *p* < 0.0001 vs. the free drug; *p* < 0.05 vs. commercial FK506 eye drops). Similar to VEGF-A and IL-6, the lowest expression of VEGFR-3 was also detected in the corneas treated with FK506 liposomes (*p* < 0.0001 vs. NS; *p* < 0.001 vs. the free drug). The free drug had no significant effect on reducing VEGFR-3 or MMP-9 (*p* > 0.05 vs. NS), while FK506 liposomes significantly reduced the expressions of the four cytokines in the corneas (*p* < 0.05 vs. NS).

CoNV is attributed to the imbalance between the expressions of pro-angiogenic factors and anti-angiogenic factors in the cornea. Among the cytokines that promote the formation of CoNV, VEGF is the most direct and is one of the strongest angiogenesis-promoting cytokines known in current research. Furthermore, VEGF-A plays a critical role in promoting neovascularization in the cornea and retina. Meanwhile, VEGFR-3 could also promote neovascularization by regulating lymphangiogenesis. In addition, angiogenesis requires the degradation of the extracellular matrix surrounding capillaries. MMPs can lyse the vascular basement membrane, promote the movement of vascular endothelial cells to the injured sites, and assist in the formation of blood vessels. MMP-2 and MMP-9, as the most effective metalloproteinases, are closely related to CoNV (Nicholas and Mysore, 2021). In addition, inflammation is also a core part of neovascularization (Roshandel et al., 2018). Research has shown that IL-6 is important in VEGF-mediated ocular neovascularization. The expression of IL-6 can be strongly induced in the early stage of corneal alkali burns (Tian et al., 2021). IL-6 can promote the expression of VEGF (Tian et al., 2021), and these two factors lead to the increase in vascular permeability and the formation of new blood vessels. These results indicate that FK506 liposomes may inhibit CoNV and inflammation by blocking the pathways related to VEGF, MMP, and IL-6.

### 3.7 Retention of FK506 Liposomes on the Ocular Surface

**FIGURE 4:**
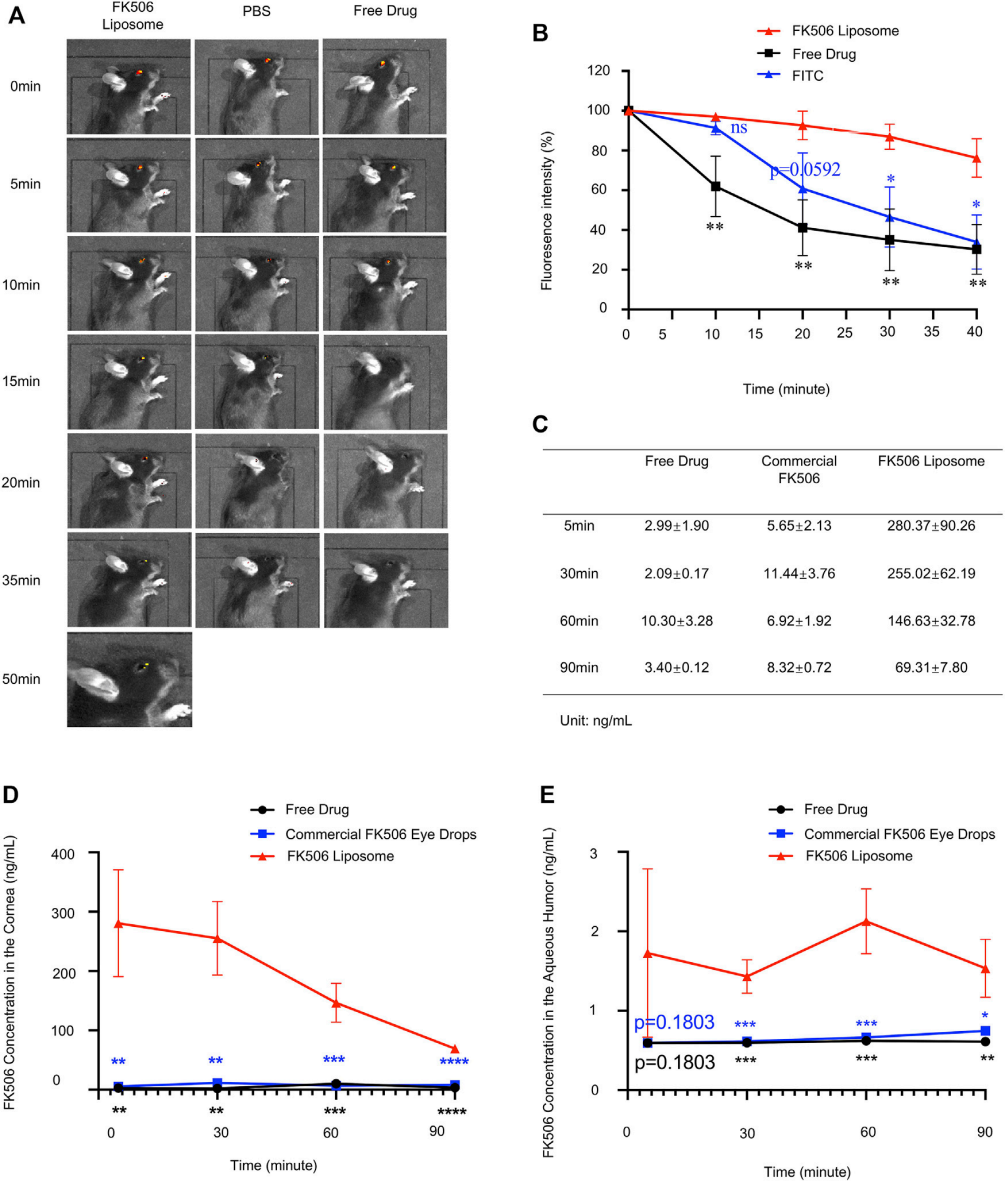
**(A)** After instilling three different preparations for 0, 5, 10, 15, 20, 35, and 50 min, the fluorescence intensity on the ocular surface. **(B)** Fluorescence intensity variation curve of different treatment groups tracked by IVIS at different time intervals (*n* = 3). **(C)** FK506 concentration in the rabbit corneas after using different FK506 preparations at different time intervals (*n* = 3). **(D)** Corneal FK506 concentration-time curve (*n* = 3); **(E)** FK506 concentration in the aqueous humor of rabbits after using different FK506 preparations at different time intervals (*n* = 3). The data was presented as mean ± SD, one-way ANOVA, *p < 0.05, **p < 0.01, ***p < 0.001, and ****p < 0.0001; ns denotes no significance.

The pre-corneal retention time was expressed as the intensity of the residual fluorescence on the ocular surface. Figure 4A shows the residual fluorescence on the ocular surface at different time points after using three different preparations. After using the eye drops for 10 min, the residual fluorescence intensity in the mice treated with FITC-labeled FK506 liposomes (97.02 ± 1.71%) was significantly higher than that in the mice treated with the free-drug solution (61.97 ± 15.3%) (*p* < 0.01). The fluorescence intensities of the free-drug solution and the fluorescent dye solution showed significant attenuation in 10–20 min and completely disappeared at the end of the 20th minute. However, at the end of the 50th minute, fluorescent signals from the mice in the cationic FK506 liposome group were still detectable. Figure 4B shows the percentage of the residual fluorescence intensity to the initial fluorescence intensity at different time points.

This finding indicated that compared with the other two preparations, cationic FK506 liposomes were not quickly eliminated from the cornea; this might be attributed to the electrostatic effect between the cationic liposomes and the negatively charged mucin layer, causing the liposomes to remain on the corneas for a longer period of time (Han et al., 2020a). Moreover, the viscosity of the FK506 liposomes was higher than those of the other two formulations; this might also play a role in prolonging the retention time on the ocular surface.

### 3.8 FK506 Content in the Cornea and the Aqueous Humor

Figures 4C,D show the concentrations of FK506 in the corneas after topical administration of three different preparations. At 5, 30, 60, and 90 min after administration, the corneal drug concentration in the FK506 liposome group was 93.77 (*p* < 0.01), 120.30 (*p* < 0.01), 14.24 (*p* < 0.001), and 20.36 times (*p* < 0.0001) higher, respectively, than in the free-drug group at the same time points. Moreover, the corneal drug level was 49.58 (*p* < 0.01), 21.95 (*p* < 0.01), 21.19 (*p* < 0.001), and 8.33 times (*p* < 0.0001) higher than that of the commercial FK506 eye drops at the time points mentioned above.

We also measured the drug content in the aqueous humor and found that the drug contents in the commercial FK506 eye drop group and the free-drug group were almost negligible at all time points. At 5, 30, 60, and 90 min after administration, the drug concentration in the aqueous humor of the FK506 liposome group was 2.91, 2.40 (*p* < 0.001), 3.43 (*p* < 0.001), and 2.51 times (*p* < 0.05) higher, respectively, than in the free-drug group at the same time points. Moreover, the cornea drug level was 2.91, 2.33 (*p* < 0.001), 3.21 (*p* < 0.001), and 2.05 times (*p* < 0.01) higher, respectively, than in the commercial FK506 eye drop group. Figure 4E illustrates the curve of the FK506 concentration in the aqueous humor over time.

The underlying mechanism of the stronger corneal permeability of FK506 liposomes might be attributed to the following aspects. First, FK506 liposomes adhered to the corneal surface and continuously penetrated the aqueous humor due to the electrostatic interaction between the positive liposomes and the negative ocular surface mucins. Second, the positively charged liposome surface assisted in opening the tight junctions between cells and increasing the absorption of the drug. Finally, due to the amphiphilic nature of liposomes and the membrane fusion between the liposomes and the corneal epithelium, hydrophobic FK506 could break through the hydrophilic and lipophilic barriers between different layers of the cornea after being encapsulated by liposomes. Commercial FK506 eye drops are an eye suspension that can retain the drug particles in the precorneal cavity to prolong the contact time between FK506 and the cornea. However, the particle size of the therapeutic agent determines the time required for the drug molecule to enter the corneal tissues, which might be the reason why the bioavailability of commercial FK506 eye drops was affected (Gote et al., 2019). Therefore, both the ocular surface retention time and corneal penetration experiments proved that cationic FK506 liposomes had a longer residence time and better corneal permeability.

## 4 Conclusion

In the present study, we demonstrated the potential of cationic FK506 liposomes in improving the ocular bioavailability and therapeutic effects of hydrophobic FK506. Compared with the free drug (0.2 mg/ml) and commercial FK506 eye drops (1 mg/ml), cationic FK506 liposomes (0.2 mg/ml) exhibited a 2.5-fold prolonged ocular residence time and an approximately 120-fold amount of the drug entering into the cornea. Furthermore, both *in vitro* and *in vivo* experiments proved that cationic FK506 liposomes had good biocompatibility. Additionally, the inhibitory effects of FK506 liposomes on VEGF-induced vascular endothelial cell migration and tube formation were also confirmed *in vitro*. Meanwhile, the present research showed that compared with the free drug and commercial FK506 eye drops, cationic FK506 liposomes could better inhibit the formation of CoNV caused by alkali burns, reduce inflammation, and promote corneal epithelial healing. Collectively, cationic FK506 liposomes might have broad prospects in the treatment of CoNV and corneal inflammation in the future.

## Data Availability

The original contributions presented in the study are included in the article/Supplementary Material, further inquiries can be directed to the corresponding authors.

## References

[B1] Alvarez-Trabado J. Diebold Y. Sanchez A. (2017). Designing Lipid Nanoparticles for Topical Ocular Drug Delivery. Int. J. Pharmaceutics 532 (1), 204–217. 10.1016/j.ijpharm.2017.09.017 28893582

[B2] Anderson C. Zhou Q. Wang S. (2014). An Alkali-Burn Injury Model of Corneal Neovascularization in the Mouse. J. Vis. Exp. 86, 51159. 10.3791/51159 PMC4164081 24748032

[B3] Chen L. Zhong J. Li S. Li W. Wang B. Deng Y. (2018). The Long-Term Effect of Tacrolimus on Alkali Burn-Induced Corneal Neovascularization and Inflammation Surpasses that of Anti-vascular Endothelial Growth Factor. Drug Des. Devel Ther. 12, 2959–2969. 10.2147/DDDT.S175297 PMC6140698 30254425

[B4] Chen Z. Mao X. Ye X. Li S. Wu T. Wang Q. (2021). A Novel and Biocompatible Nanofiber of VEGF Peptide for Enhanced Corneal Neovascularization Suppression. Chem. Eng. J. 416, 129081. 10.1016/j.cej.2021.129081

[B5] Chowdhury S. Guha R. Trivedi R. Kompella U. B. Konar A. Hazra S. (2013). Pirfenidone Nanoparticles Improve Corneal Wound Healing and Prevent Scarring Following Alkali Burn. PLoS One 8 (8), e70528. 10.1371/journal.pone.0070528 23940587 PMC3734236

[B6] Chuang Y.-L. Fang H.-W. Ajitsaria A. Chen K.-H. Su C.-Y. Liu G.-S. (2019). Development of Kaempferol-Loaded Gelatin Nanoparticles for the Treatment of Corneal Neovascularization in Mice. Pharmaceutics 11 (12), 635. 10.3390/pharmaceutics11120635 PMC6955892 31795237

[B7] Gai X. Cheng L. Li T. Liu D. Wang Y. Wang T. (2018). *In Vitro* and *In Vivo* Studies on a Novel Bioadhesive Colloidal System: Cationic Liposomes of Ibuprofen. AAPS PharmSciTech 19 (2), 700–709. 10.1208/s12249-017-0872-4 28971375

[B8] Gote V. Sikder S. Sicotte J. Pal D. (2019). Ocular Drug Delivery: Present Innovations and Future Challenges. J. Pharmacol. Exp. Ther. 370 (3), 602–624. 10.1124/jpet.119.256933 31072813

[B9] Han H. Gao Y. Chai M. Zhang X. Liu S. Huang Y. (2020a). Biofilm Microenvironment Activated Supramolecular Nanoparticles for Enhanced Photodynamic Therapy of Bacterial Keratitis. J. Controlled Release 327, 676–687. 10.1016/j.jconrel.2020.09.014 32920078

[B10] Han H. Yin Q. Tang X. Yu X. Gao Q. Tang Y. (2020b). Development of Mucoadhesive Cationic Polypeptide Micelles for Sustained Cabozantinib Release and Inhibition of Corneal Neovascularization. J. Mater. Chem. B 8 (23), 5143–5154. 10.1039/d0tb00874e 32420566

[B11] Joung C. Noh H. Jung J. Song H. Y. Bae H. Pahk K. (2020). A Novel CD147 Inhibitor, SP-8356, Attenuates Pathological Fibrosis in Alkali-Burned Rat Cornea. Int. J. Mol. Sci. 21 (8), 2990. 10.3390/ijms21082990 PMC7215672 32340317

[B12] Jumelle C. Gholizadeh S. Annabi N. Dana R. (2020). Advances and Limitations of Drug Delivery Systems Formulated as Eye Drops. J. Controlled Release 321, 1–22. 10.1016/j.jconrel.2020.01.057 PMC7170772 32027938

[B13] Kather J. N. Kroll J. (2014). Transgenic Mouse Models of Corneal Neovascularization: New Perspectives for Angiogenesis Research. Invest. Ophthalmol. Vis. Sci. 55 (11), 7637–7651. 10.1167/iovs.14-15430 25425566

[B14] Li Q. Li Z. Zeng W. Ge S. Lu H. Wu C. (2014). Proniosome-derived Niosomes for Tacrolimus Topical Ocular Delivery: *In Vitro* Cornea Permeation, Ocular Irritation, and *In Vivo* Anti-allograft Rejection. Eur. J. Pharm. Sci. 62, 115–123. 10.1016/j.ejps.2014.05.020 24905830

[B15] Lin S. Ge C. Wang D. Xie Q. Wu B. Wang J. (2019). Overcoming the Anatomical and Physiological Barriers in Topical Eye Surface Medication Using a Peptide-Decorated Polymeric Micelle. ACS Appl. Mater. Inter. 11 (43), 39603–39612. 10.1021/acsami.9b13851 31580053

[B16] Lin X. Wu X. Chen X. Wang B. Xu W. (2021). Intellective and Stimuli-Responsive Drug Delivery Systems in Eyes. Int. J. Pharmaceutics 602, 120591. 10.1016/j.ijpharm.2021.120591 33845152

[B17] Liu D. Wu Q. Chen W. Lin H. Zhu Y. Liu Y. (2019). A Novel FK506 Loaded Nanomicelles Consisting of Amino-Terminated Poly(ethylene glycol)-block-poly(D,L)-lactic Acid and Hydroxypropyl Methylcellulose for Ocular Drug Delivery. Int. J. Pharmaceutics 562, 1–10. 10.1016/j.ijpharm.2019.03.022 30878586

[B18] Mendez N. Herrera V. Zhang L. Hedjran F. Feuer R. Blair S. L. (2014). Encapsulation of Adenovirus Serotype 5 in Anionic Lecithin Liposomes Using a Bead-Based Immunoprecipitation Technique Enhances Transfection Efficiency. Biomaterials 35 (35), 9554–9561. 10.1016/j.biomaterials.2014.08.010 25154663 PMC4157089

[B19] Mu C. Shi M. Liu P. Chen L. Marriott G. (2018). Daylight-Mediated, Passive, and Sustained Release of the Glaucoma Drug Timolol from a Contact Lens. ACS Cent. Sci. 4 (12), 1677–1687. 10.1021/acscentsci.8b00641 30648151 PMC6311683

[B20] Nicholas M. P. Mysore N. (2021). Corneal Neovascularization. Exp. Eye Res. 202, 108363. 10.1016/j.exer.2020.108363 33221371

[B21] Nowak-Sliwinska P. Alitalo K. Allen E. Anisimov A. Aplin A. C. Auerbach R. (2018). Consensus Guidelines for the Use and Interpretation of Angiogenesis Assays. Angiogenesis 21 (3), 425–532. 10.1007/s10456-018-9613-x 29766399 PMC6237663

[B22] Park J.-H. Joo C.-K. Chung S. K. (2015). Comparative Study of Tacrolimus and Bevacizumab on Corneal Neovascularization in Rabbits. Cornea 34, 449–455. 10.1097/ico.0000000000000336 25651492

[B23] Pleyer U. Lutz S. Jusko W. J. Nguyen K. D. Narawane M. Rückert D. (1993). Ocular Absorption of Topically Applied FK506 from Liposomal and Oil Formulations in the Rabbit Eye. Invest. Ophthalmol. Vis. Sci. 34 (9), 2737–2742. 7688360

[B24] Qazi Y. Wong G. Monson B. Stringham J. Ambati B. K. (2010). Corneal Transparency: Genesis, Maintenance and Dysfunction. Brain Res. Bull. 81 (2-3), 198–210. 10.1016/j.brainresbull.2009.05.019 19481138 PMC3077112

[B25] Qi J. Dai R. Zhou L. Lu Y. Wu W. Liu W. (2013). Liposomes Containing Bile Salts as Novel Ocular Delivery Systems for Tacrolimus (FK506): *In Vitro* Characterization and Improved Corneal Permeation. Int. J. Nanomedicine 8, 1921–1933. 10.2147/IJN.S44487 23690687 PMC3656938

[B26] Roshandel D. Eslani M. Baradaran-Rafii A. Cheung A. Y. Kurji K. Jabbehdari S. (2018). Current and Emerging Therapies for Corneal Neovascularization. Ocul. Surf. 16 (4), 398–414. 10.1016/j.jtos.2018.06.004 29908870 PMC6461401

[B27] Sharif Z. Sharif W. (2019). Corneal Neovascularization: Updates on Pathophysiology, Investigations & Management. Rom. J. Ophthalmol. 63 (1), 15–22. 10.22336/rjo.2019.4 31198893 PMC6531773

[B28] Tian Y. Zhang F. Qiu Y. Wang S. Li F. Zhao J. (2021). Reduction of Choroidal Neovascularization via Cleavable VEGF Antibodies Conjugated to Exosomes Derived from Regulatory T Cells. Nat. Biomed. Eng. 5, 968–982. 10.1038/s41551-021-00764-3 34312509

[B29] Urtti A. (2006). Challenges and Obstacles of Ocular Pharmacokinetics and Drug Delivery. Adv. Drug Deliv. Rev. 58 (11), 1131–1135. 10.1016/j.addr.2006.07.027 17097758

[B30] Whitcup S. M. Pleyer U. Lai J. C. Lutz S. Mochizuki M. Chan C.-C. (1998). Topical Liposome-Encapsulated FK506 for the Treatment of Endotoxin-Induced Uveitis. Ocul. Immunol. Inflamm. 6 (1), 51–56. 10.1076/ocii.6.1.51.8079 9798194

[B31] Wu D. Zhao Z. Kim J. Razmi A. Wang L. L. W. Kapate N. (2021). Gemcitabine and Doxorubicin in Immunostimulatory Monophosphoryl Lipid A Liposomes for Treating Breast Cancer. Bioeng. Transl Med. 6 (1), e10188. 10.1002/btm2.10188 33532588 PMC7823124

[B32] Wu Y. Xu Z. Yang Y. Qiu J. Yang M. Wu C. (2019). Tetramethylpyrazine (TMP) Ameliorates Corneal Neovascularization via Regulating Cell Infiltration into Cornea after Alkali Burn. Biomed. Pharmacother. 109, 1041–1051. 10.1016/j.biopha.2018.10.091 30551354

[B33] Zahir‐Jouzdani F. Khonsari F. Soleimani M. Mahbod M. Arefian E. Heydari M. (2019). Nanostructured Lipid Carriers Containing Rapamycin for Prevention of Corneal Fibroblasts Proliferation and Haze Propagation after Burn Injuries: *In Vitro* and *In Vivo* . J. Cel Physiol 234 (4), 4702–4712. 10.1002/jcp.27243 30191977

[B34] Zeng W. Li Q. Wan T. Liu C. Pan W. Wu Z. (2016). Hyaluronic Acid-Coated Niosomes Facilitate Tacrolimus Ocular Delivery: Mucoadhesion, Precorneal Retention, Aqueous Humor Pharmacokinetics, and Transcorneal Permeability. Colloids Surf. B: Biointerfaces 141, 28–35. 10.1016/j.colsurfb.2016.01.014 26820107

[B35] Zhang R. He R. Qian J. Guo J. Xue K. Yuan Y.-f. (2010). Treatment of Experimental Autoimmune Uveoretinitis with Intravitreal Injection of Tacrolimus (FK506) Encapsulated in Liposomes. Invest. Ophthalmol. Vis. Sci. 51 (7), 3575–3582. 10.1167/iovs.09-4373 20164461

[B36] Zhong Y. Wang K. Zhang Y. Yin Q. Li S. Wang J. (2021). Ocular Wnt/β-Catenin Pathway Inhibitor XAV939-Loaded Liposomes for Treating Alkali-Burned Corneal Wound and Neovascularization. Front. Bioeng. Biotechnol. 9, 753879. 10.3389/fbioe.2021.753879 34765592 PMC8576519

