# Supplementary material for: Inhibition of Neovascularization and Inflammation in a Mouse Model of Corneal Alkali Burns Using Cationic Liposomal Tacrolimus
Source: Front Bioeng Biotechnol. 2021 Dec 7;9:791954. doi: 10.3389/fbioe.2021.791954 (PMC8688995; doi:10.3389/fbioe.2021.791954)
Supplement: Supplementary file 1 [file DataSheet1.docx]

Supplementary Material

## Supplementary Figures

**
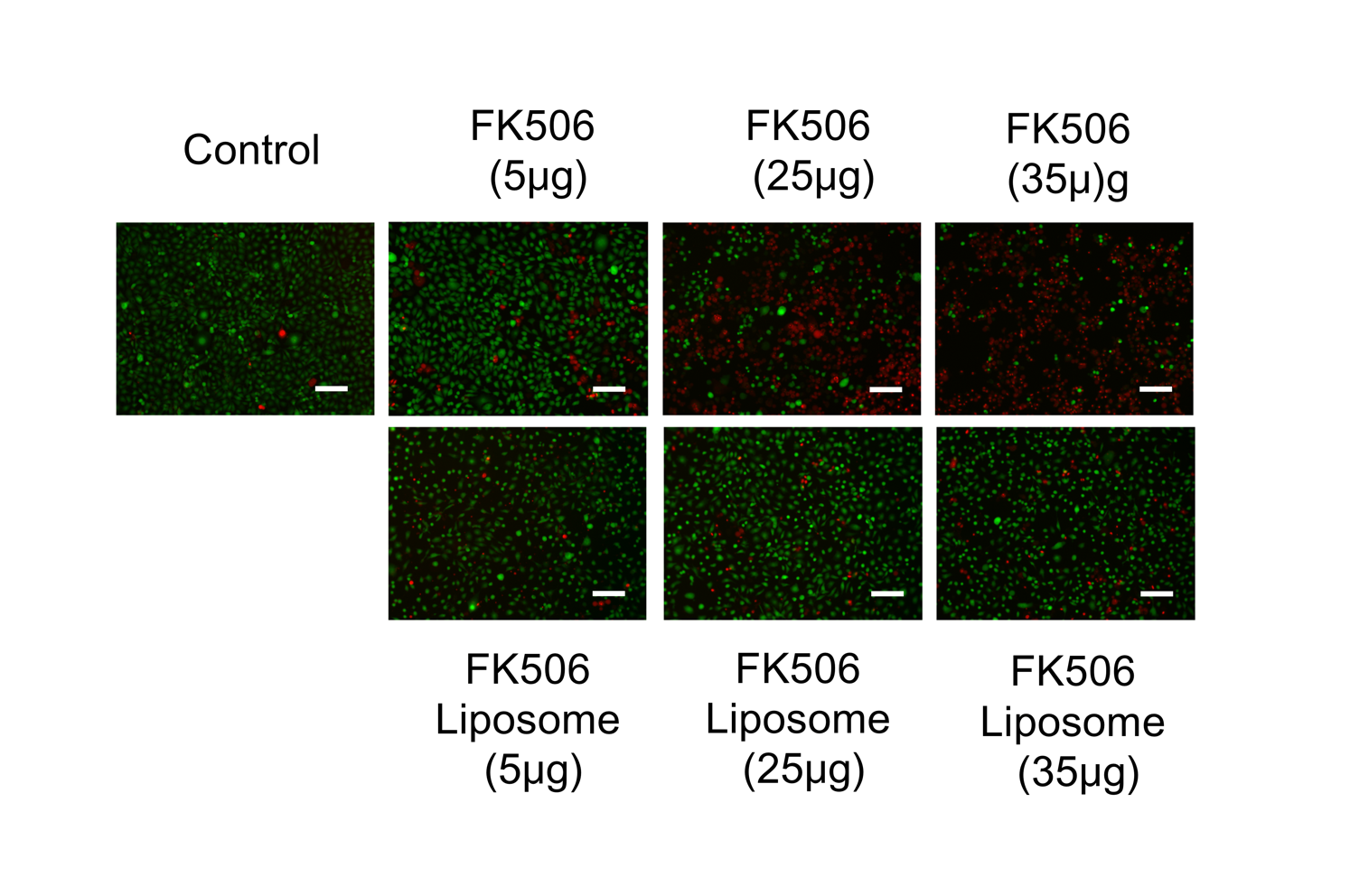
**

**Supplementary Figure 1.** Live and dead cell assay of the HCECs exposed to free-drug or FK506 liposomes at different concentrations. Scale bar: 200 μm.


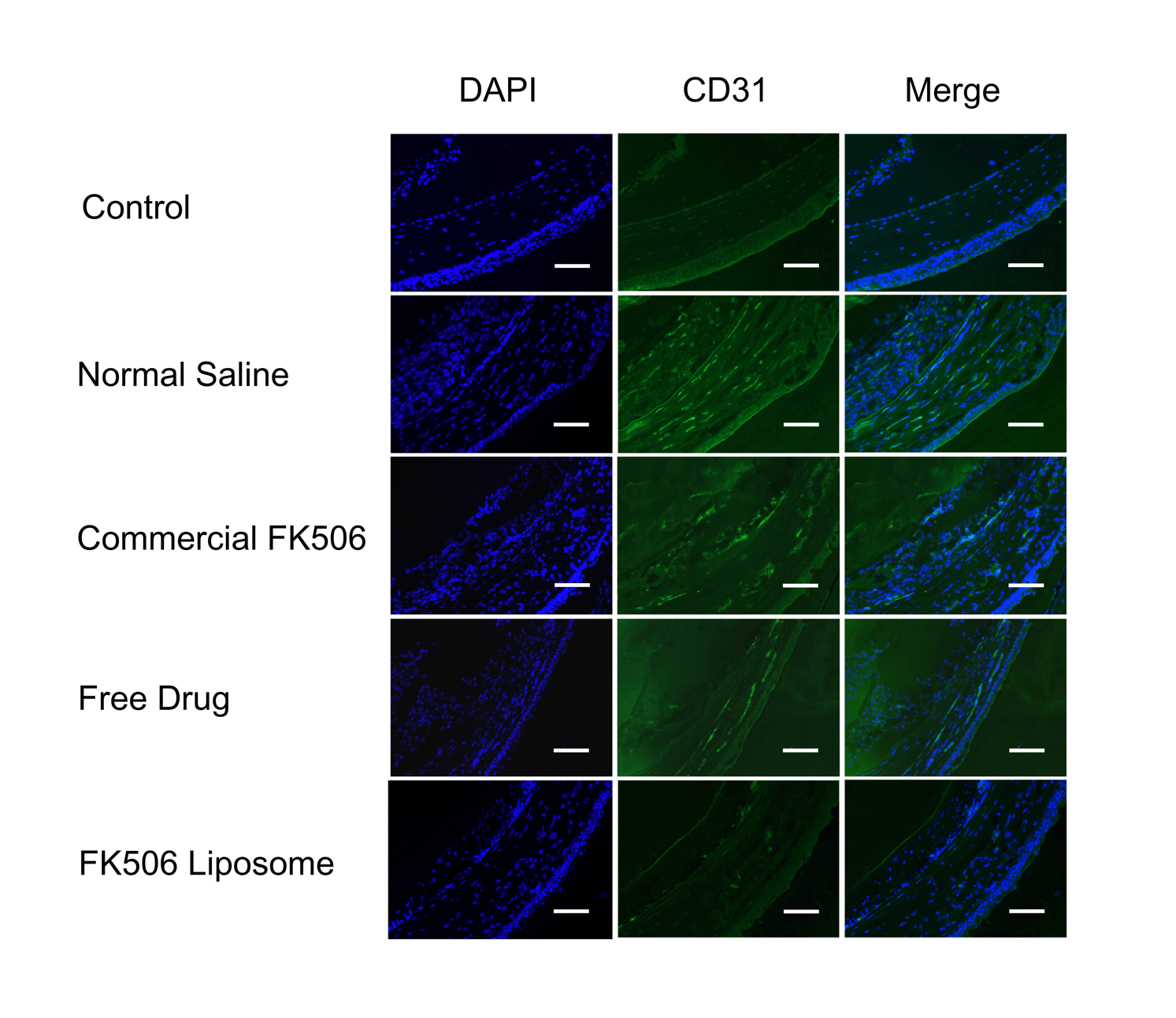


**Supplementary Figure 2.** CD31 vascular endothelial marker immunofluorescence staining of corneal sections after treatment with various formulations for 14 days. The cell nucleus was stained blue and the new blood vessel were stained green. Scale bar: 100 μm.
